# Disease features and management of cardiomyopathies in women

**DOI:** 10.1007/s10741-024-10386-x

**Published:** 2024-02-03

**Authors:** Alberto Aimo, Paolo Morfino, Chiara Arzilli, Giuseppe Vergaro, Valentina Spini, Iacopo Fabiani, Vincenzo Castiglione, Claudio Rapezzi, Michele Emdin

**Affiliations:** 1 Scuola Superiore Sant’Anna, Pisa, Italy; 2 Cardiology Division, Fondazione Toscana Gabriele Monasterio, Pisa, Italy; 3 Cardiologic Centre, University of Ferrara, Ferrara, Italy; 4 Maria Cecilia Hospital, GVM Care & Research, Cotignola (Ravenna), Ravenna, Italy

**Keywords:** Cardiomyopathies, Women, Sex, Differences, Clinical features, Outcomes, Management

## Abstract

Over the last years, there has been a growing interest in the clinical manifestations and outcomes of cardiomyopathies in women. Peripartum cardiomyopathy is the only women-specific cardiomyopathy. In cardiomyopathies with X-linked transmission, women are not simply healthy carriers of the disorder, but can show a wide spectrum of clinical manifestations ranging from mild to severe manifestations because of heterogeneous patterns of X-chromosome inactivation. In mitochondrial disorders with a matrilinear transmission, cardiomyopathy is part of a systemic disorder affecting both men and women. Even some inherited cardiomyopathies with autosomal transmission display phenotypic and prognostic differences between men and women. Notably, female hormones seem to exert a protective role in hypertrophic cardiomyopathy (HCM) and variant transthyretin amyloidosis until the menopausal period. Women with cardiomyopathies holding high-risk features should be referred to a third-level center and evaluated on an individual basis. Cardiomyopathies can have a detrimental impact on pregnancy and childbirth because of the associated hemodynamic derangements. Genetic counselling and a tailored cardiological evaluation are essential to evaluate the likelihood of transmitting the disease to the children and the possibility of a prenatal or early post-natal diagnosis, as well as to estimate the risk associated with pregnancy and delivery, and the optimal management strategies.

## Introduction

**Fig. 1:**
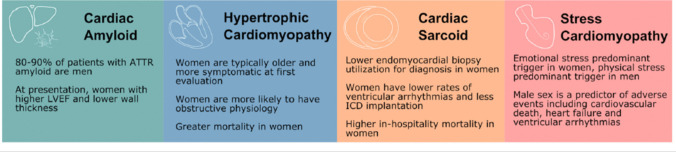
Main sex-related differences in cardiomyopathies. Although overall less data are available in women, infiltrative and restrictive cardiomyopathies as well as stress cardiomyopathy have unique sex differences. In cardiac amyloidosis, it is unclear to what extent the male predominance of the disease is due to ascertainment bias and the potential role for sex-specific diagnostic criteria. Similarly, women with hypertrophic cardiomyopathy are typically more symptomatic at first evaluation, which may be due to the use of similar wall thickness cut-offs for both sexes. ATTR, amyloid transthyretin; ICD, implantable cardioverter defibrillator; LVEF, left ventricular ejection fraction. Reprinted with permission from: DeFilippis et al. [4]

Sex-related differences in clinical presentation and outcomes have been reported for several cardiovascular diseases, particularly ischemic heart disease [1] (Fig. 1). Evidence on sex-related differences in cardiomyopathies is much more limited [2]. In this review, we will provide an up-to-date summary on the following topics: 1) specific cardiomyopathies of women, 2) genetic cardiomyopathies transmitted exclusively by women, 3) peculiar features of cardiomyopathies and cardiac parameters in women, 4) influence of maternal cardiomyopathy on pregnancy, and 5) utility of genetic counselling in women with hereditary cardiomyopathies seeking a pregnancy.

## Peripartum cardiomyopathy

The only women-specific cardiomyopathy is peripartum cardiomyopathy (PPCM), which develops from the last month of pregnancy up to 6 months after childbirth (with the highest incidence during the first month after childbirth), and manifests with dilated cardiomyopathy (DCM) and heart failure (HF) [3]. PPCM is more common in African-Americans and is strongly associated with more advanced maternal age, gestational hypertension, preeclampsia, twin pregnancy, and diabetes mellitus [4]. The 16-kDa N-terminal prolactin fragment (16KDa-PRL) produced at the end of pregnancy or shortly after childbirth seems to have a pathogenic role [5]. This fragment affects the metabolism and contractility of cardiomyocytes and promotes inflammation and apoptosis. Combination therapy with inhibitors of PRL synthesis (e.g., bromocriptine) and standard HF treatment was found to promote a left ventricular (LV) function recovery and to reduce the risk of cardiovascular outcomes in women with severe PPCM [6, 7], warranting a class IIb, level of evidence B recommendation for bromocriptine by the European Society of Cardiology (ESC) Guidelines on the management of HF during pregnancy [8]. A recent meta-analysis including 8 studies (although just 2 randomized and controlled) on 593 patients demonstrated that bromocriptine use to treat PPCM is associated with significantly higher survival (92% vs. 84%, relative risk (RR) 1.11, *p* = 0.02), although it does not seem to significantly reduce the risk of major adverse cardiovascular outcomes (13.7% vs. 33.3%, RR 0.60, *p* = 0.54) or promote LV ejection fraction (LVEF) recovery (46.9% vs. 46.8%, RR 0.94, *p* = 0.74) [9]. The Impact of Bromocriptine on Clinical Outcomes for Peripartum Cardiomyopathy (REBIRTH) trial has been designed to further investigate the effect of bromocriptine on functional recovery and outcomes in women with PPCM (NCT05180773).

## Peculiar features of cardiomyopathies in women

The two forms of cardiomyopathies for which sex-specific features have been well characterized are sarcomeric hypertrophic cardiomyopathy (HCM) and cardiac amyloidosis (CA).

**Fig. 2:**
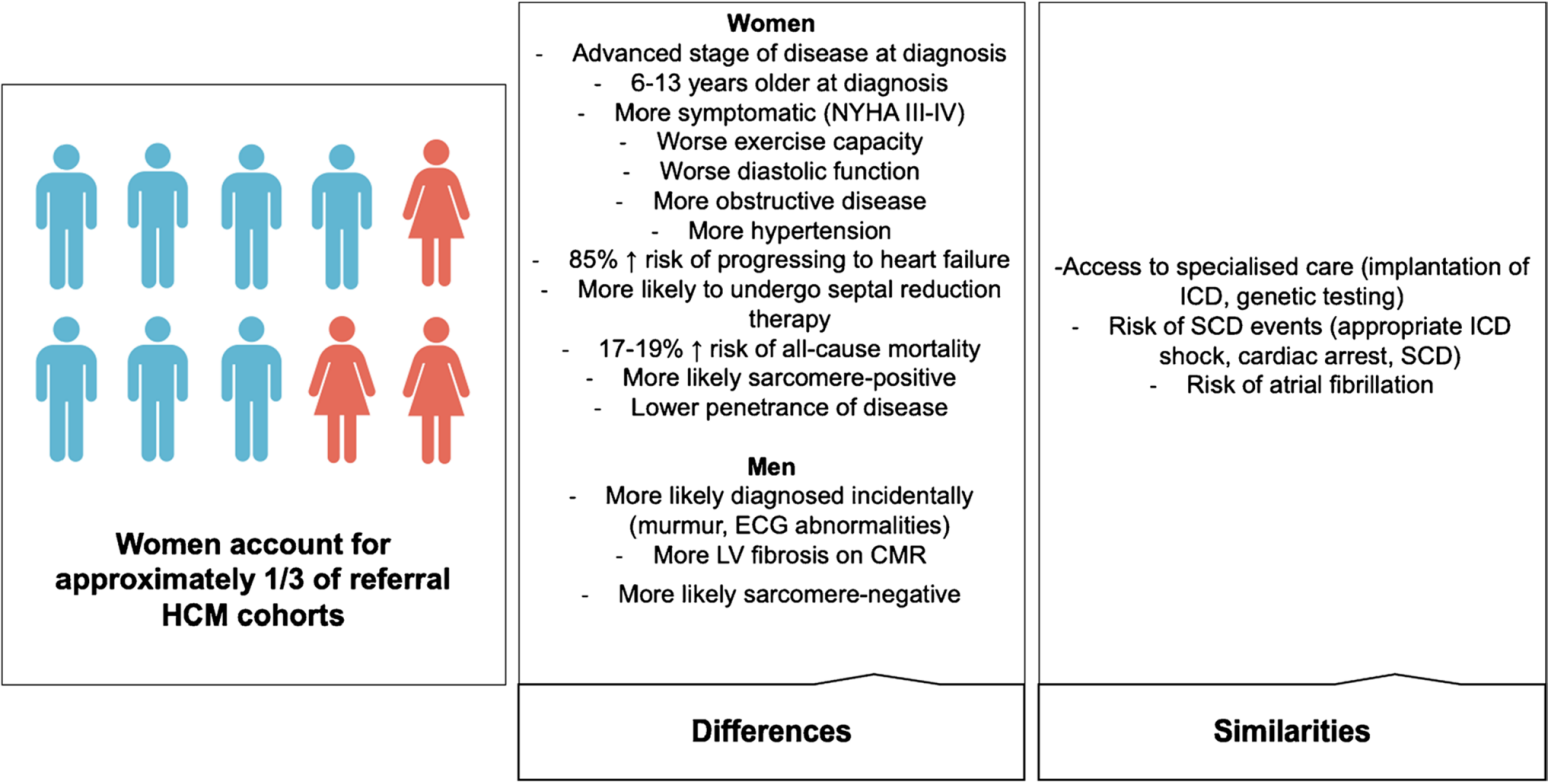
Sex differences in clinical profile, genotype, and outcomes in hypertrophic cardiomyopathy. CMR, cardiac magnetic resonance; ECG, electrocardiogram; HCM, hypertrophic cardiomyopathy; ICD, implantable cardioverter defibrillator; LV, left ventricular; NYHA, New York Heart Association; SCD, sudden cardiac death. Modified with permission from: Butters et al. [15]

**Fig. 3:**
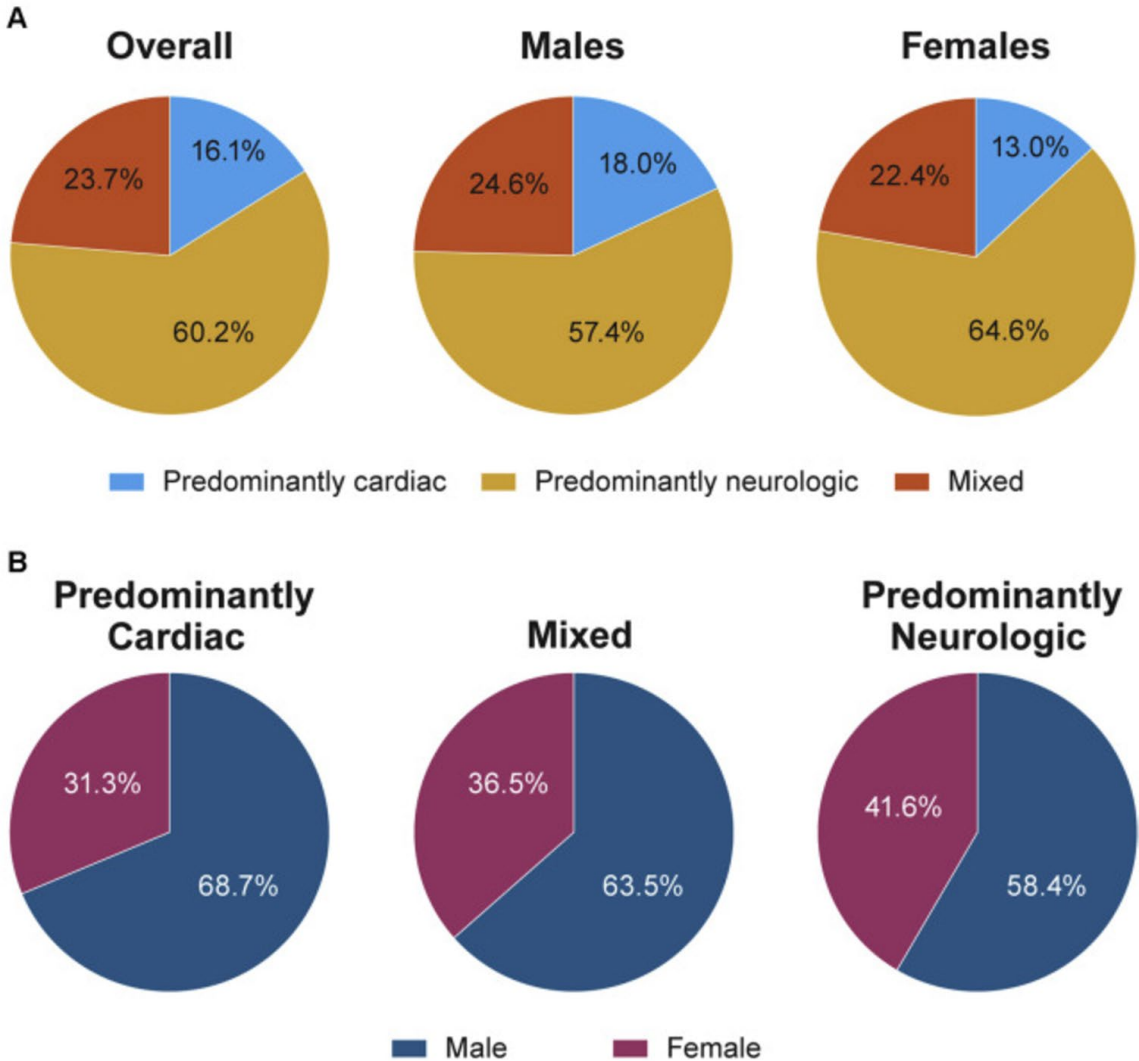
Association between sex and phenotypes in patients with variant amyloid transthyretin (ATTRv) amyloidosis. **A** Distribution of phenotype according to sex. **B** Distribution of sex according to phenotype. A predominantly cardiac phenotype was more common in men than in women (*p* < 0.001), whereas a predominantly neurologic phenotype was less common in men than in women (*p* < 0.001); similar proportions of men and women had a mixed phenotype (*p* = 0.22). Patients with ATTRv amyloidosis were those with a disease causing TTR genetic variant and symptoms definitely related to ATTR amyloidosis. Patients were grouped into a predominant clinical phenotype based on clinical presentation at enrolment. Predominantly cardiac was defined as at least 1 of the following symptoms: heart failure, dyspnea, or abnormal electrocardiogram caused by rhythm disturbance; and no more than mild neurologic or gastrointestinal symptoms (excluding erectile dysfunction, constipation, and carpal tunnel syndrome). Predominantly neurologic was defined as walking disability, other neurologic symptoms, and/or 1 of the following gastrointestinal symptoms, of any severity: early satiety, nausea, vomiting, unintentional weight loss, diarrhoea, or fecal incontinence; and without heart failure, dyspnea, or abnormal electrocardiogram caused by rhythm disturbance. Mixed was defined as all remaining patients with at least 1 of the cardiac and 1 of the neurologic symptoms as described above. Reprinted with permission from: Caponetti et al. [26]

Sarcomeric HCM is a hereditary disorder with an autosomal dominant transmission, variable phenotypic presentation, and incomplete penetrance [10]. Disease prevalence is higher in men, with a male to female ratio of 1.5 [11, 12]. Women have a higher age at diagnosis compared to men (with an average difference of 10 years); are more often symptomatic of dyspnoea, chest pain, and syncope; and hold a greater risk of dying from HF [13, 14], with a steep increase in the incidence of adverse events after the menopause (Fig. 2) [12, 15]. Nonetheless, the causes of these sex-related differences still need to be clarified. CA is a heterogeneous disease including subtypes with different pathophysiology. Amyloid light-chain (AL) amyloidosis is characterized by the overproduction of misfolding-prone immunoglobulin light chains, with cardiac involvement ranging from 50 to 75% and a slightly higher prevalence in men [4, 16, 17]. A recent study reported significant lower median values for normalized LV end-diastolic volume, stroke volume, and mean normalized LV mass in women with AL amyloidosis than in men, but no sex-related differences in the response to treatment and outcomes have emerged [18]. Amyloid transthyretin (ATTR) amyloidosis can be due to pathogenic mutations in the *TTR* gene (variant ATTR, ATTRv) or be an age-related phenomenon (wild-type ATTR, ATTRwt). Patients with ATTRv may have a cardiac or neurologic disease or both, while those with ATTRwt show almost exclusive cardiac involvement [19]. ATTR-CA manifests typically as LV (pseudo)hypertrophy and/or HF with preserved ejection fraction, possibly accompanied by conduction disturbances and/or arrhythmias. Men account for around 80% of patients with ATTRwt, and around 70% of those with ATTRv [20–25]. In a systematic review assessing 4669 patients with ATTR-CA, 791 (17%) were women, including 174 (9%), 366 (29%), and 251 (18%) in studies on ATTRwt-CA, ATTRv-CA, and undefined ATTR-CA, respectively [22]. In 2790 patients with ATTRv from the Transthyretin Amyloidosis Outcomes Survey (THAOS), male patients (59% of the whole cohort) were more likely to have symptoms of cardiac involvement and a cardiac phenotype. Male prevalence was greater in patients with more severe cardiac manifestations of disease, as assessed with N-terminal pro–B-type natriuretic peptide, LVEF, LV wall thickness, and LV mass index (Fig. 3). Male sex, age at disease onset, and genotype category emerged as independent predictors of cardiac disease [26]. The hypothesis regarding the potential protective role of female sex hormones on myocardial involvement was supported by one study showing that women with ATTR-CA and prominent cardiac involvement were more likely to be postmenopausal [27]. Moreover, some cardiac structural and functional differences have been reported: women have a lower interventricular septal and posterior wall thickness and LV end-diastolic diameter, and a higher LVEF, compared to men across different ATTR-CA subtypes [22]. Since LV wall thickness ≥ 12 mm is the suggested threshold for ATTR-CM diagnosis in both sexes [28], smaller cardiac anatomy in women with the disease may lead to underdiagnosis of early-stage disease. Data on sex-related differences in outcomes of ATTR patients are still scarce. No major differences in all-cause mortality and survival have been identified between men and women with ATTR amyloidosis [18, 22]. Single-center studies suggested comparable outcomes after cardiac transplantation between men and women with ATTR, AL, and nonamyloid-related cardiomyopathy [29].

Sex-related differences in the prevalence and clinical manifestations have been reported also for sarcoidosis, a systemic inflammatory disorder characterized by non-caseating granulomas located mostly in the lungs and thoracic lymph nodes [30]. A study on ethnic differences in patients with sarcoidosis showed that African-American women aged between 30 and 39 years have a greater incidence of the disease than age-matched African-American men, and also age-matched Caucasian men and women. African-American women are younger at the time of diagnosis and are more often symptomatic and with more advanced disease (in terms of extra-thoracic involvement) [31]. Cardiac disease is found in 20 to 30% of patients, without significant sex-related differences, and is characterized by granulomas located mostly within the interventricular septum, conduction disturbances, and arrhythmias.

There are limited data on the influence of female sex on the phenotypic expression of DCM, arrhythmogenic cardiomyopathy (AC), and LV non-compaction, although there are some reports of a greater prevalence of these conditions among men [32–34].

Takotsubo cardiomyopathy (TC) is an acquired and reversible cardiomyopathy usually occurring after an emotionally or physically stressful event, especially in postmenopausal women [4, 11, 12]. TC has a much higher prevalence in women, with a female to male ratio of 9:1. The diagnostic algorithms for TC include the Mayo Clinic Criteria and the International Expert Consensus Document on Takotsubo Syndrome, both of which consider female sex as a risk factor [13, 14]. The pathophysiology of TC and the reason why women are more often affected are not completely understood. Estrogen deficiency and intense catecholamine response to emotional stimuli might play a role [15]. Physical stress is a more common trigger in men, who also have a lower mean age at presentation, and a higher risk of developing severe LV systolic dysfunction or requiring mechanical circulatory support than women [4, 16, 17].

## Genetic cardiomyopathies transmitted exclusively by women

X-linked and matrilinear inheritance are women-specific modes of transmission of genetic cardiomyopathies. An X-linked transmission is found in the following cardiovascular conditions: Anderson-Fabry disease, Danon, disease, Hunter syndrome, and dystrophinopathies. Conversely, some mitochondrial disorders have a matrilinear transmission. Table 1 reports the transmission pattern and the men-to-women ratio of the main cardiomyopathies grouped according to phenotypes proposed in 2023 guidelines [35].

**Table 1:** Transmission modality and approximate ratio of prevalence in the two sexes of the main cardiomyopathies

**Cardiomyopathy**	**Modality of transmission**				**M/W ratio**
	AD	AR	X-linked	Matrilinear	
Sarcomeric HCM	X				3:2
Anderson-Fabry disease (HCM)			X		3:2
Danon disease (HCM)			X		1:1
Hunter disease (HCM)			X		N/A
Amyloidosis (RCM) AL amyloidosis ATTRv amyloidosis ATTRwt amyloidosis	X				1:1 3:2 9:1
Friederich disease (HCM)			X		1:1
Noonan disease (HCM)	X				1:1
Leopard disease (HCM)	X				1:1
Mitochondriopathies (HCM or DCM) Mithocondrial DNA mutation Nuclear DNA mutation	X	X	X	X	1:1 1:1
Emery-Dreifuss muscular dystrophy type 1 (DCM)			X		N/A
Emery-Dreifuss muscular dystrophy type 2 (DCM)	X				1:1
Lamin A/C mutations (DCM or NDLVC)	X				1:1
Filamin C mutations (NDLVC)	X				N/A
Phospholamban mutations (NDLVC)	X				N/A
Transmembrane protein 43 mutations (NDLVC)	X				N/A
RNA-binding motif protein 20 mutations (NDLVC)	X				N/A
Dystrophinopathies (DCM) Duchenne muscular dystrophy Becker muscular dystrophy Isolated DCM			X X X		N/A N/A N/A
Myotonic dystrophy type 1 and 2 (DCM)	X				N/A
Limb-Girdle muscular dystrophy (DCM)	X	X			N/A
Desminopathies (DCM or NDLVC)	X	X			2:1
ARVC	X	X			3:1

*AD* autosomal dominant, *AL* amyloid light-chain, *AR* autosomal recessive, *ARVC* arrhythmogenic right ventricular cardiomyopathy, *ATTR* amyloid transthyretin, *CM* cardiomyopathy, *DCM* dilated cardiomyopathy, *HCM* hypertrophic cardiomyopathy, *M/W* male to women, *NDLVC* non-dilated left ventricular cardiomyopathy, *RCM* restrictive cardiomyopathy

Modified with permission from: Biagini et al. [2]

The phenotypic expression of women heterozygous for an X-linked mutation depends on the pattern of casual inactivation of one of the two X-chromosomes in somatic cells (lyonization) [36]. Nevertheless, women usually manifest with milder signs and symptoms, a later onset, slower progression, and longer survival than men [2].

Anderson-Fabry disease, Hunter syndrome, and Danon disease are X-linked disorders characterized by an enzymatic defect preventing the degradation of specific substances which end up accumulating into lysosomes, ensuing in the involvement of several organs including the heart. Anderson-Fabry disease is caused by defective alpha-galactosidase activity determining a lysosomal accumulation of glycosphingolipids [37]. In men, the clinical spectrum is wide and includes acroparesthesia, hypo-anhidrosis, angiokeratomas, and, starting from the second decade of life, renal failure, and cardiac manifestations [38]. The most common sign in female carriers is corneal dystrophy (70% of cases), which can be the only disease manifestation. Cardiac involvement typically presents with LV hypertrophy, with a male-to-female ratio of 1.5, and a 10-year later onset in women [39, 40]. Permanent and paroxysmal atrial fibrillation (AF) have an incidence of 14% and 4% in men and women, respectively; non-sustained ventricular tachycardia episodes are more common in men (8%) [41]. The measurement of enzyme activity has a diagnostic role to confirm or discard the disease only in men, while the diagnosis in women requires the search for the genetic mutation, as the enzymatic deficiency can be mild or undetectable [42].

Danon disease is caused by a deficit of the lysosome-associated membrane protein 2. Typical manifestations are skeletal myopathy, intellectual disability, and heart disease. Cardiac involvement has a similar prevalence in men and women and usually consists of concentric LV hypertrophy, with an evolution towards DCM in later stages [43]. Men usually die within the third decade, mostly of cardiac causes. Women have a later disease onset and a lower degree of intellectual disability, and cardiomyopathy can be the main disease manifestation [44, 45].

Hunter syndrome is the only mucopolysaccharidosis with X-linked transmission, thus occurring almost exclusively and more severely in males [46]. It is caused by defective iduronate-2-solphatase activity. Patients with Hunter syndrome shows a wide spectrum of multisystemic clinical symptoms and the involvement of the heart leads to cardiomyopathy and valvular disease. In the most serious cases, death occurs within the first or second decade of life, often due to obstructive respiratory disease or heart failure [46]. Disease severity in women is usually mild to moderate, with mild dysmorphic features, dysostosis, and cardiovascular disease. Patients often have arterial hypertension, valve heart disease (most commonly mitral regurgitation and aortic stenosis), and LV hypertrophy. Isolated right ventricular involvement has been reported [47, 48]. Women can survive in discrete conditions until the adult age and sometimes, in the mildest forms, even after the sixth decade [2]. The prenatal diagnosis can be made by measuring foetal iduronate-2-sulphatase activity in the plasma, chorionic villi, or amniotic liquid cells, with the caveat that enzymatic activity can be slightly reduced or normal in female fetuses [49].

Dystrophin is expressed both in the skeletal muscle and in the heart, with the main function of stabilizing the cellular membrane by connecting the cytoskeleton to the extracellular matrix. Mutations in the dystrophin gene cause Duchenne muscular dystrophy (DMD), Becker muscular dystrophy (BMD), and isolated cardiac disease (manifesting as DCM) [50, 51]. DMD and BMD are characterized by absent or reduced dystrophin expression, respectively, causing progressive degeneration and fibro-fatty replacement in the skeletal muscle. Cardiac involvement usually manifests in the form of DCM, which develop in almost all adult patients.

Patients are typically males, with symptom onset during the first decade of life in DMD and in the second decade for BMD and isolated DCM. In patients with DMD, death is usually caused by respiratory insufficiency, pulmonary infections, and HF, usually occurring around the third decade of life [50]. In women carrying DMD or BMD mutations, the clinical phenotype ranges from asymptomatic to invalidating forms based on the specific lyonization pattern, and DCM may often represent the only manifestation. Disease onset is usually in the adult age, although isolated cases of cardiac disease before adolescence have been reported [52].

Mitochondrial disorders are characterized by defective functioning of the respiratory chain, whose proteins are codified either by the nuclear or the mitochondrial DNA. Mutations in the mitochondrial genes have a matrilineal inheritance and are characterized by a functional impairment of tissues mostly depending on oxidative metabolism, such as the skeletal muscle, the central nervous system, the retina, the kidneys, and the heart [53]. The main cardiac manifestations of mitochondrial disorders are atrioventricular conduction disturbances, ventricular pre-excitation, and HCM without outflow tract obstruction; DCM is less common [53].

Diabetic women can transmit a non-genetic form of cardiomyopathy that consists of a transient LV (pseudo)hypertrophy often already evident on foetal ultrasound scans [54]. The underlying pathogenic causes are maternal hyperglycemia and the resulting foetal hyperinsulinism determining an accumulation of glycogen in the heart. This condition is usually found when the mother has decompensated diabetes and is usually reversible within 2 to 12 months from birth [55]. Some cases of increased wall thickness, particularly the interventricular septum, have been reported also in foetuses of women with well-controlled diabetes [56].

## Systolic and diastolic function in men versus women

In healthy individuals, the female heart shows smaller volumes and mass compared to men, even considering values normalized for body surface area. Women have also a higher stroke volume despite adjustment for end-diastolic volume (EDV) and other potential confounders. Many studies conducted on general population reported a higher LVEF for women compared to men [57–60], and sex-specific reference values for cardiovascular magnetic resonance studies have been proposed [61]. The difference between men and women in normal LVEF values is much less prominent than in LV volumes and mass. Nonetheless, this difference may become relevant in several settings, for example the diagnosis of mild DCM, the assessment of systolic function in the background of restrictive cardiomyopathies [62], and in the cut-off levels for LVEF to recommend selected interventions [63].

Transthoracic echocardiography is the gold standard for the analysis of diastolic function, which is comparable in the two sexes, so that sex-specific reference values are not required [64].

## Influence of maternal cardiomyopathies on pregnancy

Deep knowledge of the physiological changes in the cardiovascular system during pregnancy, labor, and delivery is needed to understand the possible impact of cardiomyopathies. An increase in cardiac output is needed to allow greater perfusion of the uterus [65]. Such increase is obtained through a rise in stroke volume and an expansion of the blood pool, as well as (from the 20th week onwards) an increase in heart rate [66, 67]. The cardiac chambers enlarge while LV systolic function remains unchanged [8]. Blood pressure decreases following the reduction of peripheral resistances due to the development of placental circulation and the vasodilation sustained by local mediators, such as nitric oxide, prostacyclins, and endothelium-dependent factors [66, 67]. Prominent hemodynamic fluctuations are observed during the labor and delivery. Pain and anxiety induce catecholamine release and tachycardia, and uterine contraction and compression of the inferior vena cava by the uterus cause a redistribution of blood volume from uterine vessels, placenta, and lower limbs, increasing cardiac preload [67]. During labor, cardiac output increases by 20% [66]. Delivery is associated with a significant blood loss (about 500 mL for a vaginal delivery and 1000 mL for caesarean delivery) and is followed by a rapid increase in peripheral resistances. Hemodynamic function usually normalizes some weeks after delivery [68].

Women with cardiomyopathy assuming contraception should be addressed with safe medications. Among contraceptives, ethinyloestradiol is associated with the higher risk of thromboembolic events and should be avoided [69]. On the other side, progestin or levonorgestrel and intrauterine contraceptive devices are well tolerated. Patients receiving artificial insemination may experience ovarian hyperstimulation syndrome, which increases the risk of thrombosis. Finally, hormonal stimulation may be contraindicated in patients with HCM or ventricular tachycardia not receiving anticoagulation therapy [35].

### Hypertrophic cardiomyopathy

The physiological changes associated with pregnancy are normally well tolerated by women with HCM [8]. For example, blood volume expansion and increased LV end-diastolic volume can reduce the dynamic outflow tract obstruction [2]. Beta-blockers and calcium channel blockers can be continued without risks for the foetus, as growth retardation and bradycardia have been rarely reported [8]. The adaptation to labor and delivery is more problematic, and great attention to the balance between fluids and diuretics is warranted [2]. The reduced filling time because of tachycardia, the progressive increase in blood volume, and cardiac output in a small heart with reduced compliance can impair the diastolic filling of the LV. Furthermore, the Valsalva manoeuvre and blood loss can exacerbate outflow tract obstruction [2]. In 199 pregnancies of 100 women with HCM, 2 deaths for arrhythmic causes during the delivery were reported [70]. The risk of sudden cardiac death (SCD) seems limited to women with important risk factors already present before the pregnancy, such as a prominent interventricular septal hypertrophy with severe outflow tract obstruction, whereas asymptomatic patients have a low risk of complications. In 271 pregnancies of 127 women with HCM, a low incidence of complications was found, with just 2 cases of HF during delivery, and no deaths [71].

Overall, pregnancy is usually well-tolerated in women with HCM and vaginal delivery might be considered [8]. Nonetheless, women who are symptomatic despite medical therapy or have severe outflow tract obstruction should be evaluated at a tertiary referral centre and managed by a multidisciplinary team [8]. Adequate analgesia is recommended to reduce the catecholaminergic state induced by pain, but epidural anaesthesia causes systemic vasodilation and hypotension and should then be used with caution in patients with severe outflow tract obstruction [8]. There are no clear indications on the management of delivery; hence, the choice must be individualized based on the clinical phenotype. In patients with HF, a cesarean delivery should be preferred [8].

### DCM

The increase in blood volume caused by pregnancy and the rise in afterload during labor and delivery may alter the labile hemodynamic equilibrium of patients with LV contractile dysfunction. In women with DCM, appropriate counselling is strongly recommended before planning a pregnancy, because of the high risk of irreversible deterioration in ventricular function, maternal mortality, and fetal loss [8]. The main risk factors associated with a poor prognosis are moderate or severe LV dysfunction (LVEF < 40%), New York Heart Association class III or IV symptoms, and a previous cardiovascular event. When one of these risk factors is present, the likelihood of HF decompensation, most often during the third trimester and after childbirth, is about 60% [72]. Even the risk of neonatal complications is strongly influenced by these 3 factors. The risk is particularly high when LVEF is < 20%, mitral regurgitation, right ventricular failure, AF, and/or hypotension are present [8]. Furthermore, if LVEF falls during pregnancy, a reconsideration of the safety of pregnancy is advised [8].

The management of medical therapy is challenging because clinicians have limited options. Angiotensin-converting enzyme inhibitors and angiotensin receptor blockers are contraindicated because of the possible teratogenic effects on the fetal kidney [73, 74]. There is no sufficient data on the negative effects of mineralocorticoid receptor antagonists, which are then contraindicated and their use in normokalaemic patients with well-controlled or mild hypertension should be carefully discussed [75]. Sacubitril/valsartan and sodium-glucose cotransporter-2 inhibitors should not be prescribed as their teratogenic potential is unknown. Even loop diuretic therapy is discouraged because of the risk of placental hypoperfusion and should be prescribed only in cases with severe peripheral or pulmonary congestion [76]. Conversely, beta-blockers are relatively safe. Among the beta-blockers for hypertension in pregnancy, labetalol is the recommended choice, whereas unselective beta-blockers such as atenolol have been associated with a higher risk of fetal growth retardation [8, 77–79].

ESC guidelines do not provide specific recommendations on the modality of delivery in DCM [8]. The choice must be individualized based on the functional status of the patient and the fetus growth, following a discussion between multiple specialists and the mother. Vaginal delivery is associated with less blood loss and a lower risk of infection, venous thrombosis, and embolism. Vaginal delivery might be contraindicated when the mother has severe disease features or is on vitamin K antagonists, due to the risk of foetal intracranial bleeding [8, 80].

### Arrhythmogenic cardiomyopathy

AC is a disorder with an autosomal dominant transmission pattern characterized by fibro-fatty replacement of the myocardium in one or both ventricles predisposing to ventricular arrhythmias and SCD [81–84]. Evidence about the possible complications of pregnancy in women with AC is still limited. In a study conducted on 6 patients who had AC with mild or moderate ventricular dysfunction and receiving antiarrhythmic therapy, pregnancy and vaginal delivery resulted well tolerated. However, patients enrolled showed a significant propensity to develop arrhythmias during the last trimester and the postpartum [85]. A recent retrospective study analyzed 648 live births from 692 pregnancies in 367 women with AC. Results suggested that the risk of adverse events during pregnancy, labor, and postpartum were similar in AC women on pregnancy compared with AC women without a history of pregnancy [86]. In other small case series, pregnancy and postpartum did not increase mortality and complications compared to women with AC who had not given birth [86–89].

### Restrictive cardiomyopathies

Restrictive cardiomyopathies are rare and heterogeneous conditions characterized by a preserved or slightly reduced LV systolic function and severe diastolic dysfunction [90]. The increase in blood volume during pregnancy might theoretically increase the risk of pulmonary edema in patients with restrictive physiology, but the few case reports available seem not to support this hypothesis [91, 92].

### Anticoagulation therapy during pregnancy

Cardiac arrhythmias, especially AF, are frequently detected in patients with cardiomyopathies and proper anticoagulation therapy is required [93]. The administration of vitamin K antagonists (VKA) during the first trimester of pregnancy is associated with a risk of congenital disorders (e.g., limb defects and hypoplastic nasal bone) up to 10% [94, 95]. VKA should also be avoided before vaginal delivery because fetal intracranial bleeding represents a possible complication. Therefore, the replacement of VKA with heparin is recommended during weeks 6–12 and the periodical monitoring of international normalized ratio (INR) should be weekly performed [35, 96]. Novel oral anticoagulants are not recommended during pregnancy and breastfeeding, but their safety profile is still unknown [97–99].

## Utility of genetic counselling in women of childbearing age with hereditary cardiomyopathies

Genetic counselling is essential for women with genetic cardiomyopathies seeking a pregnancy [100]. This should include an assessment of the expected penetrance and the implications on the health and quality of life of affected children. Prenatal diagnosis might be considered in relatively rare cases where a specific mutation has been found in the mother. There are no clear guidelines regulating prenatal genetic testing. The mother should be informed that the presence of a mutation does not necessarily imply an inevitable disease development and that disease severity depends on the combination of genetic, epigenetic, and environmental factors. Prenatal diagnosis through genetic testing in cardiomyopathies is then not recommended and should be decided on an individual basis, except for specific disorders or high-risk situations, which need to be discussed by expert teams after detailed clinical and family assessment [8, 100].

## Conclusions

Sex-related differences in patients with cardiomyopathies have been explored in a limited number of studies. The few data available support a protective role of female hormones until menopause in both HCM and ATTR amyloidosis. The identification of a biological substrate may lead to the identification of novel therapeutic targets such as prolactin in peripartum cardiomyopathy. Furthermore, the tools of molecular biology allow confirmation or discard of specific disorders and extend the analysis to family members. A more accurate phenotypic characterization has led to understanding that women are not simply healthy carriers of pathogenic mutations, but also can show a wide spectrum of clinical manifestations ranging from mild to severe manifestations because of heterogeneous patterns of X-chromosome inactivation. Because most cardiomyopathies have a genetic basis, adequate genetic counselling at a referral centre is advisable for women seeking pregnancy. Genetic counselling and a tailored cardiological evaluation are essential to evaluate the likelihood of transmitting the disease to the children and the possibility of a prenatal or early post-natal diagnosis, to estimate the risk associated with pregnancy and delivery, and the optimal management strategies. Finally, we may envisage greater attention to sex-related differences in the phenotypic expression and outcomes of cardiomyopathies, the identification of more accurate models for risk prediction, and the identification of safe and effective therapies for women with systolic dysfunction.

## Data Availability

Not applicable.

## Abbreviations

AC: Arrhythmogenic cardiomyopathy
AF: Atrial fibrillation
AL: Amyloid light-chain
ATTR(v/wt): Amyloid transthyretin (variant/wild-type)
BMD: Becker muscular dystrophy
CA: Cardiac amyloidosis
DCM: Dilated cardiomyopathy
DMD: Duchenne muscular dystrophy
EF: Ejection fraction
ESC: European Society of Cardiology
HCM: Hypertrophic cardiomyopathy
HF: Heart failure
LV: Left ventricular
LVEF: Left ventricular ejection fraction
PPCM: Peripartum cardiomyopathy
PRL: Prolactin
SCD: Sudden cardiac death
TC: Takotsubo cardiomyopathy
VKA: Vitamin K antagonists
